# Poster Session II - A220 A SYSTEMATIC REVIEW OF DEFINITIONS AND DIAGNOSIS OF CROHN’S DISEASE COLONIC STRICTURES

**DOI:** 10.1093/jcag/gwaf042.220

**Published:** 2026-02-13

**Authors:** C Lu, R Dhaliwal, A Kellar, C Rowan, J St-Pierre, K E Suarez, M O’Brien, R E Rosentreter, V Gulhati, M E Baker, D Bruining, J Fletcher, I Gordon, V Jairath, B Feagan, F Rieder

**Affiliations:** Medicine, University of Calgary, Calgary, AB, Canada; Medicine, University of Calgary, Calgary, AB, Canada; The University of Chicago Comer Children’s Hospital, Chicago, IL; Beaumont Hospital, Dublin, Leinster, Ireland; Medicine, University of Calgary, Calgary, AB, Canada; Universidad de Costa Rica, San José, San José Province, Costa Rica; Medicine, University of Calgary, Calgary, AB, Canada; Medicine, University of Calgary, Calgary, AB, Canada; Medicine, University of Calgary, Calgary, AB, Canada; Cleveland Clinic, Cleveland, OH; Mayo Clinic Minnesota, Rochester, MN; Mayo Clinic Minnesota, Rochester, MN; Cleveland Clinic, Cleveland, OH; Medicine, Western University, London, ON, Canada; Medicine, Western University, London, ON, Canada; Cleveland Clinic, Cleveland, OH

## Abstract

**Background:**

Colonic strictures in Crohn’s disease (CD) are an important, but understudied clinical problem. Cross-sectional imaging modalities such as computed tomography (CT), magnetic resonance imaging (MRI), and intestinal ultrasound (IUS) effectively assess the small bowel in CD allowing for the diagnosis of strictures. However, radiologic definitions of colonic strictures and their diagnostic accuracy have not been developed. Endoscopic definitions of colonic strictures are the most well-described.

**Aims:**

We aimed to systematically review available data to summarize definitions, diagnosis and differentiation between inflammation and fibrosis of colonic strictures on endoscopy, CT, MR, and IUS

**Methods:**

We conducted a systematic review following PRISMA guidelines using MEDLINE, Scopus, CINAHL, and CENTRAL databases from inception to Oct 6, 2024 to identify literature with diagnostic imaging that included patients with colonic CD strictures. To distinguish fibrosis from inflammation, only studies comparing endoscopy or imaging to a full thickness histologic gold standard were included. Two stage screening was completed in duplicate, and risk of bias was assessed using QUADAS-2

**Results:**

46 studies were eligible for inclusion. Definitions for colonic strictures on CT, MR, and IUS are heterogeneous with combinations of bowel wall thickness, luminal narrowing, and pre-stenotic dilation with no clear cut offs. Endoscopic definitions commonly included lack of colonoscope passage through a narrowed lumen. The diagnostic accuracy of colonic strictures on imaging to detect inflammation and fibrosis against a gold standard of endoscopy or surgical resection is unclear as direct comparisons are few. Meta-analyses were not conducted due to methodological heterogeneity of grading fibrosis and statistical heterogeneity of sensitivity, specificity, and accuracy of colonic stricture diagnoses limited by pooling with small bowel strictures in the studies

**Conclusions:**

This systematic review describes a lack of definitions for the diagnosis of CD colonic strictures particularly on diagnostic imaging, and the limitations with the current available data to differentiate inflammation from fibrosis. Although bowel wall thickening, luminal narrowing, and pre-stenotic dilation are recognized as hallmark features of small bowel strictures, their adaptation to the colon requires further assessment. Definitions for colonic strictures should likely include imaging combined with endoscopy. Future endeavors are required to develop a consensus to define and diagnose colonic strictures on these modalities

**Funding Agencies:**

None

